# Antituberculosis Drug Resistance Survey in Lesotho, 2008-2009: Lessons Learned

**DOI:** 10.1371/journal.pone.0133808

**Published:** 2015-07-24

**Authors:** Llang B. Maama-Maime, Mathabo Mareka, Julia V. Ershova, Thabong E. Tlali, Kekeletso Kao, Mamakhetha Phalatse, Lauren Polansky, Laura K. Beres, Moselinyane Letsie, Timothy H. Holtz

**Affiliations:** 1 National TB Programme, Ministry of Health, Maseru, Lesotho; 2 Laboratory Services, Ministry of Health, Maseru, Lesotho; 3 Centers for Disease Control and Prevention, Atlanta, United States of America; 4 Emory University, Atlanta, United States of America; 5 Disease Control, Ministry of Health, Maseru, Lesotho; Universidad Nacional de La Plata., ARGENTINA

## Abstract

**Setting:**

Drug resistance is an increasing threat to tuberculosis (TB) control worldwide. The World Health Organization advises monitoring for drug resistance, with either ongoing surveillance or periodic surveys.

**Methods:**

The antituberculosis drug resistance survey was conducted in Lesotho in 2008-2009. Basic demographic and TB history information was collected from individuals with positive sputum smear results at 17 diagnostic facilities. Additional sputum sample was sent to the national TB reference laboratory for culture and drug susceptibility testing.

**Results:**

Among 3441 eligible smear-positive persons, 1121 (32.6%) were not requested to submit sputum for culture. Among 2320 persons submitted sputum, 1164 (50.2%) were not asked for clinical information or did not have valid sputum samples for testing. In addition, 445/2320 (19.2%) were excluded from analysis because of other laboratory or data management reasons. Among 984/3441 (28.6%) persons who had data available for analysis, MDR-TB was present in 24/773 (3.1%) of new and 25/195 (12.8%) of retreatment TB cases. Logistical, operational and data management challenges affected survey results.

**Conclusion:**

MDR-TB is prevalent in Lesotho, but limitations reduced the reliability of our findings. Multiple lessons learned during this survey can be applied to improve the next drug resistance survey in Lesotho and other resource constrained countries may learn how to avoid these bottlenecks.

## Introduction

Drug-resistant tuberculosis (DR-TB) threatens global TB control and is a major public health concern in many countries. Multidrug-resistant tuberculosis (MDR-TB) and Extensively Drug-Resistant tuberculosis (XDR-TB) are increasingly being found in resource-limited settings [1–2]. Globally, 136 412 cases of MDR-TB or rifampicin-resistant TB (RR-TB) who were eligible for MDR-TB treatment were notified to World Health Organization (WHO) in 2013, mostly by countries in the European Region, India and South Africa. Furthermore, in South Africa, DR-TB and HIV have converged in a deadly syndemic, defined by increased incidences of TB and HIV, endemic transmission of DR-TB strains, high mortality rates, and poor treatment outcomes [3–4]. About 10% of MDR-TB cases in South Africa have XDR-TB [5].

**Fig 1:**
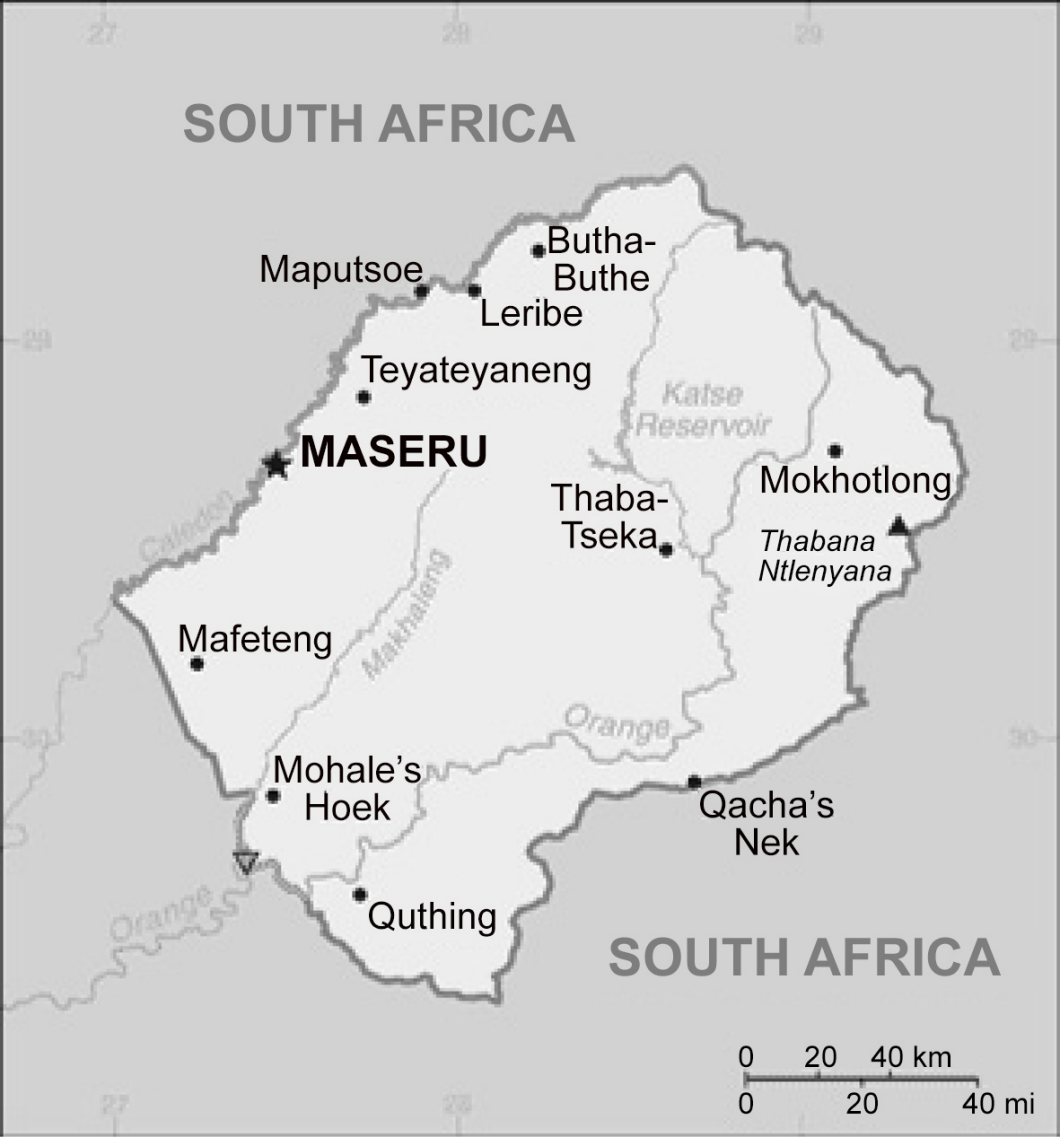
Map of Lesotho. Image source: https://www.cia.gov/library/publications/the-world-factbook/index.html.

The Kingdom of Lesotho (Lesotho) is a mountainous country completely surrounded by South Africa (Fig 1). Mean temperature ranges are -3°C to 32°C in the lowlands and -8.5°C to 20°C in high altitude areas. Frost occurrence can last between February and November in the high altitude areas [6]. The WHO reported population of 2.1 million people living in Lesotho in 2013. The average life expectancy at birth in 2013 was 48.7 years, a reduction from global norms that is largely attributable to the HIV/AIDS epidemic in the country [7]. The prevalence of HIV among adults 15–49 years old has stabilized at a high level and has not significantly decreased since the year 2000; currently prevalence of HIV stands at 23% [1, 7]. TB remains a major challenge to public health in the country. Lesotho is among the 21 countries with the highest incidence of TB, reporting 916 new TB cases per 100,000 population in 2013 [1].

A national MDR/XDR-TB program in Lesotho was started in collaboration with partners in 2007, followed by upgrading laboratory capacity to diagnose TB and the establishment of an MDR/XDR-TB referral Hospital in Botsabelo [8]. According to the drug resistance survey conducted in Lesotho in 1995, 12.3% of all TB cases had any form of resistance, 11% had isoniazid resistance, and 1.6% had rifampin resistance [9]. However, the high MDR-TB prevalence (10%) and the outbreak of XDR-TB in the neighboring KwaZulu-Natal Province of South Africa have generated serious concerns as to the real extent of the MDR-TB problem in Lesotho [10–11].

In 2008, the National Tuberculosis Program (NTP), supported by partners, conducted a drug resistance survey (DRS) to determine the extent and pattern of TB drug resistance in the country. This manuscript presents the main findings of the DRS in Lesotho, discusses the logistical, laboratory and other operational challenges encountered during the survey and provides recommendations for future surveys.

## Methods

The Lesotho NTP conducted a cross-sectional survey on TB drug resistance involving sputum smear microscopy, culture, and drug susceptibility testing (DST) among newly diagnosed smear-positive pulmonary TB patients from 23 June 2008 to 31 March 2009. The study was designed to conform to WHO protocol guidelines for a periodic drug resistance survey [11]. Based on the number of new smear-positive TB cases in Lesotho and available data on rifampin resistance in neighboring South Africa, the estimated sample size for the survey was 896 cultures from new smear-positive patients to give a precision within 1%–2% of the true value with 95% confidence. All smear-positive retreatment patients diagnosed during the survey period were included in the survey but did not contribute to the calculated sample size.

Every patient ≥ 15 years-old seen at inpatient and outpatient sites during the survey period who had a sputum sample submitted to any of the 17 national TB diagnostic centers, received a smear-positive result and a diagnosis of pulmonary TB was eligible for the survey. Patients, incarcerated at the time of the survey, residents of mental health institutions, and those already receiving TB treatment were excluded. Personnel of all 17 diagnostic centers in Lesotho were instructed to record the demographic information and the results from three sputum smears on an eligibility form and the treatment history on a clinical form for all cases during the survey period. Each eligible patient was assigned a unique identification number (ID) and asked to submit an additional sample to be sent to the Lesotho National Tuberculosis Reference Laboratory (NTRL) for culture. The forms were transported to the NTP office in Maseru where data were entered into two standardized EpiData databases: the ‘Eligibility’ and ‘Clinical’ databases. HIV data were not included in this survey, however all patients were offered HIV Testing and Counselling as part of comprehensive package of care and those who turn positive could access treatment.

Lowënstein-Jensen (LJ) cultures for the survey specimens were performed in the NTRL. DST was not done in Lesotho because proficiency testing had not been completed by the time of the survey. Culture-positive isolates were sent from the NTRL to a Supranational Reference Laboratory (SRL) in Borstel, Germany for DST; the results were entered in the ‘Laboratory’ database. The survey was intended to continue until the required sample size was reached.

We related Eligibility and Clinical databases using survey unique ID for data analysis. CDC staff assisted in data management and validation including finding discrepancies in the data, tracking down missing records, determining patient’s DST results, and finalizing the database for analysis. Study staff attempted to locate the original forms, correct the discrepancies, and match records. In cases where we could not identify the original form, an algorithm based on patient’s age, sex, district, diagnostic center, and date of registration was used to match a patient’s clinical information to their eligibility information.

To assess the limitations identified during data analysis, we evaluated the survey’s standard operating procedures (SOPs) including flow of specimen and logistics, laboratory records and other information related to data accuracy and completeness.

### Ethics Statement

The study does not involve human subjects above and beyond what is normally conducted during routine medical care for TB suspects, namely the collection of sputum for examination. CDC and the Lesotho NTP determined this activity as public health surveillance rather than human subjects research. A formal written waiver for the need of ethics approval was issued by CDC/DTBE associate director of science.

## Results

**Fig 2:**
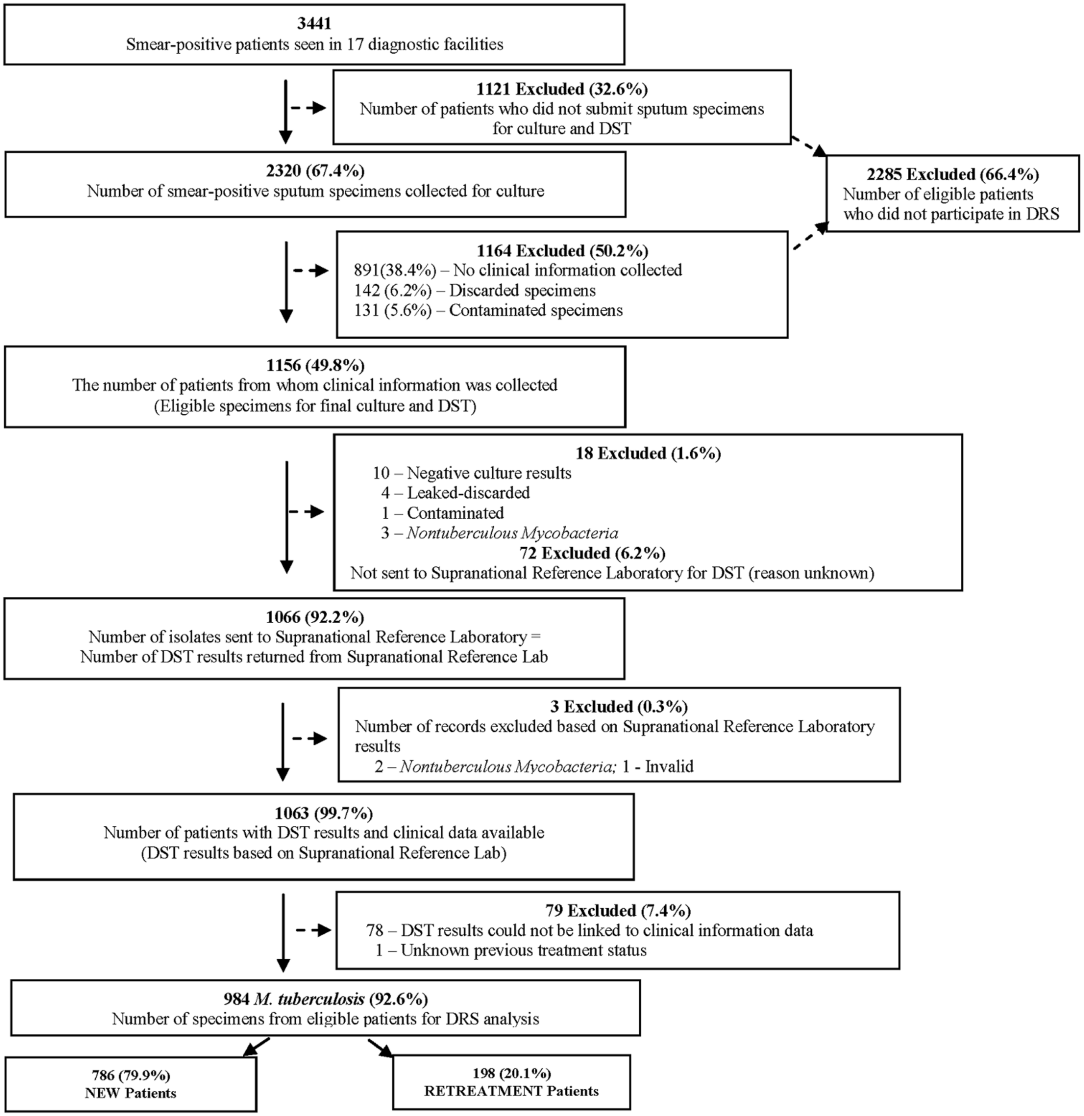
Lesotho Drug Resistance Survey (DRS) Sampling Flow Diagram.

The national TB services registered 3441 smear-positive persons with pulmonary TB at 17 diagnostic centers during the survey period (June 2008–March 2009). Sputum specimens for culture were collected from 2320 (67.4%) persons (Fig 2). However, 1164 (50.2%) of the 2320 eligible persons who submitted sputum specimens for culture were excluded from the study for the following reasons: 142 (6.2%) had specimens that were discarded because of labeling errors, 131 (5.6%) had specimens that were contaminated, and 891 (38.4%) persons had no clinical information collected (Fig 2). The remaining 1156 (49.8%) persons had both sputum available for culture and demographic and clinical information available for analysis.

Among these 1156 persons, 18 were further excluded from analysis: 10 had no growth on culture, four had specimen cups that leaked and were discarded, one had culture contamination, and three cultures grew non-tuberculosis mycobacteria (NTM). The remaining 1138 persons had positive *M*. *tuberculosis* cultures; among them cultures from 1066 were sent to Borstel SRL for first-line DST. Laboratory testing in the Borstel SRL excluded an additional three persons for the following reasons: two with specimens that grew NTM only and one patient‘s culture was determined not viable. After data review, an additional 79 cases were excluded from analysis because study data could not be matched with DST results (n = 78) and one had no treatment history recorded. The final database for analysis included 984 cases with both complete data and *M*. *tuberculosis* DST results from the Borstel SRL, or 28.6% of 3441 eligible persons during the survey period. Among 984 cases in the final database, 786 (79.9%) were new. Main demographic characteristics were similar between 984/3441 individuals with complete data and 2457/3441 individuals who were eligible but did not participate in the survey. Median age was 35 years old in both groups; 58.4% and 57.5% were males in each group, respectively.

We found that 81/773 (10.5%) of new TB patient isolates in Lesotho had *M*. *tuberculosis* isolates resistant to at least isoniazid or rifampin and 3.1% of new TB patients had MDR-TB (Table 1). Among retreatment patients, 23.1% had isolates resistant to at least isoniazid or rifampin: 16.4% had any resistance to isoniazid and 19.5% had any resistance to rifampin. Mono-resistance to either isoniazid or rifampin occurred in 8.7% of isolates among retreatment patients, while 12.8% of retreatment patients had MDR-TB. The patterns of resistance to other first-line drugs (streptomycin, ethambutol, pyrazinamide) and key second-line drugs for MDR-TB isolates are presented in Table 2.

**Table 1:** First-line drug susceptibility test results, Lesotho, 2008–2009 (N = 984).

**New Patients**	**n = 786** ^†^
*Resistance to isoniazid or rifampin*	81 (10.5%)
isoniazid	57 (7.4%)
rifampin	48 (6.2%)
*Monoresistance*	44 (5.7%)
isoniazid	33 (4.3%)
rifampin	13 (1.7%)
*Multidrug resistance*	24 (3.1%)
**Previously Treated Patients**	**n = 198** ^§^
*Resistance to isoniazid or rifampin*	45 (23.1%)
isoniazid	32 (16.4%)
rifampin	38 (19.5%)
*Monoresistance*	17 (8.7%)
isoniazid	7 (3.6%)
rifampin	10 (5.1%)
*Multidrug resistance*	25 (12.8%)
**All Patients**	**n = 984** ^†^ ^§^
*Resistance to isoniazid or rifampin*	126 (13.0%)
isoniazid	89 (9.2%)
rifampin	86 (8.9%)
*Monoresistance*	61 (6.3%)
isoniazid	40 (4.1%)
rifampin	23 (2.4%)
*Multidrug resistance*	49 (5.1%)

^†^Missing results—new patients with missing isoniazid result = 13

^§^Missing results—previously treated patients with missing isoniazid result = 3

**Table 2:** First and second-line drug susceptibility test results for MDR-TB isolates, Lesotho, 2008–2009 (N = 39).

**New Patients**	**n = 19**
*Any other 1* ^*st*^ *line drug resistance*	16 (84.2%)
Any streptomycin	16 (84.2%)
Any ethambutol	12 (63.2%)
Any pyrazinamide	11 (57.9%)
*Any 2* ^*nd*^ *line drug resistance*	11 (57.9%)
Any ethionamide	11 (57.9%)
Any cycloserine	0 (0.00%)
Any ofloxacin	0 (0.00%)
Any para-amino salicylic acid	0 (0.00%)
Any amikacin	1 (5.3%)
Any capreomycin	1 (5.3%)
**Previously Treated Patients**	**n = 20** *
*Any other 1* ^*st*^ *line drug resistance*	15 (85.0%)
Any streptomycin	15 (75.0%)
Any ethambutol	11 (55.0%)
Any pyrazinamide	12 (63.2%)
*Any 2* ^*nd*^ *line drug resistance*	10 (50.0%)
Any ethionamide	10 (50.0%)
Any cycloserine	0 (0.00%)
Any ofloxacin	1 (5.3%)
Any para-amino salicylic acid	0 (0.0%)
Any amikacin	1 (5.3%)
Any capreomycin	1 (5.3%)

*Missing results—previously treated with missing amikacin = 1; capreomycin = 1; cycloserine = 1; ethionamide = 1; ofloxacin = 1; para-amino salicylic acid = 1; pyrazinamide = 1

MDR-TB—multidrug-resistant tuberculosis.

Upon careful investigation of logistical, laboratory and other information collected during survey implementation, we determined that three obvious breakdowns in procedures detracted from this survey.

### 1. Logistics

We found that 1121/3441 (32.6%) eligible patients had missing specimens because patients were being seen but no sputum samples for DRS were being collected. We also found that 1164 (50.2%) of 2320 patients with sputum samples collected for DRS had missing clinical information. We later determined that this happened because the specimen for DRS was not requested during the patient’s first visit to the diagnostic facility. If a patient did not return, no effort was made to follow up with the patient; thus, the specimen for DRS was never collected. According to personnel at the diagnostic centers, patients were not coming back for various reasons including difficult or expensive transportation and bad weather (snow). Furthermore, clinical forms were not completed for some patients who came back to provide an additional sputum sample for DRS. Specific logistical problems that were reported included the shortages in supply of data collection forms, the courier not collecting samples on time, and labels not being attached properly to specimen containers.

### 2. Laboratory

The NTRL reported many missing specimens. The main reported reason for missing specimens was the logistical issues encountered during survey implementation. The other laboratory challenges included low sample volumes and leakage of samples during transportation. Winter weather hazards were also a consideration, blocking transport for specimens. In addition, crystallization of the transport medium for sputum samples occurred because of cold weather.

### 3. Data management

A large number of the records in the ‘Clinical’ database did not have a corresponding record in the ‘Eligibility’ database. This was most often because of inconsistency in assignment of the DRS unique ID and recording sex on the respective Clinical and Eligibility forms. The majority of the Clinical forms with corresponding missing Eligibility forms came from five diagnostic centers (Maseru, Berea, Leribe, Motebeng and Mafeteng) for patients who were registered at the start of the survey. The study coordinator reported that during data collection at these sites, clinical forms were sometimes completed before eligibility forms, increasing the probability of missing forms and inconsistent data recording.

Inconsistencies in assignment of the DRS unique ID resulted in a substantial portion of first-line DST results not matching with patients’ clinical information. Because of this, 13% of patients’ first-line DST results were excluded from analysis. In some cases we were able to find patient laboratory numbers in district hospital records, and this allowed for abstraction of the missing clinical information. Other data management issues included missing data in the clinical history sections, dates written incorrectly, and skip patterns on the data collection forms that were not followed.

## Discussion

The implementation of this drug resistance survey in Lesotho was complicated, and the reliability of the results was diminished by survey problems in logistics, laboratory, and data management. Clinical and laboratory information required for the survey analysis was collected for only one third (984/3441) of eligible individuals, including 786 new TB cases. The estimated sample size of 896 cultures from new smear-positive cases was not reached.

Large nation-wide studies such as drug resistance survey in resource limited settings are challenging [12–14]. Laboratory capacity was reported as the most operational barrier for many surveys [14]. Other reported operational barriers included the considerable human resources needed to interview patients and verify classification, and the extensive national and international transport networks required to ship sputum specimens, cultures, and *M*. *tuberculosis* isolates within and across national borders [12]. Some desirable and valuable components of surveys—for example, larger sample sizes, better differentiation of subcategories of previously treated cases, HIV testing and DST to second-line drugs—come at great additional expense and workload. In addition, survey data are prone to errors that may to some extent invalidate the findings. Errors, or biases, may be related to selection of subjects, laboratory testing, and data collection or analysis [12].

Lesotho faced these barriers despite the intention to conduct the survey as part of country capacity building. The main weakness of this survey was the large proportion of eligible TB patients seen during the survey period that was not included in the survey either because a sputum sample for DRS was not submitted or clinical information was not collected (66.4%). The major reason for losing eligible patients was waiting to collect an additional specimen for DRS and clinical information until the patent came back for microscopy results. It appeared to be limited to specific locales which could be targeted for special training and more innovative approaches the next time a survey is conducted. In some instances patients could not come back due to harsh terrain and weather as that was a year of severe winter with snow in Lesotho. Another weakness of the survey was failure to collect HIV data. Due to the high HIV prevalence in the country, surveillance of HIV among MDR-TB over time is important and should be included in the survey operations [5,12]. Laboratory challenges including specimen’s transportation failures and crystallization of sputum samples were due to winter weather and could be addresses by better organization of sputum transportation during winter seasons. Others laboratory issues including low sample volumes, leakage of samples during transportations, and mislabeling of the tubes for sputum collection were associated with lack or deficiency of training for nurses and related DRS personnel in local health centers. Refreshing trainings along with intensive supervision and monitoring during DRS implementation should be conducted for all levels of the field operations. Appropriate instructions and available training materials for patients at the enrollment facilities could also improve quality and provide the required amount of sputum for the survey. The main reason of losing first-line DST results during the survey was inconsistency in assignment of DRS unique ID to enrolled patients. At some enrollment sites, a local treatment registration number was used as a DRS ID, resulting in losing eligible patients that did not come back for treatment. Clear instructions for data collection and management should be provided prior the survey implementation.

Nonetheless, our findings suggested the possibility of prevalent DR-TB in new patients in Lesotho (Table 1). Efforts by the Government of the Kingdom of Lesotho should be aimed at rapid case detection, thorough drug susceptibility testing, timely initiation of treatment, and infection control measures in TB and HIV treatment clinics in order to interrupt transmission. In addition, the isolates from MDR-TB patients showed resistance to other first-line drugs that were tested in the Borstel laboratory. Although few MDR isolates were tested, the prevalence of resistance to streptomycin and ethambutol was worrisome (Table 2). First-line drugs are recommended for the treatment of MDR-TB whenever the DST results indicate susceptibility [13]. A high degree of resistance to all first-line drugs will make treatment of MDR-TB more difficult for the national program.

We could not draw conclusions about the prevalence of second-line drug resistance, and thus the prevalence of XDR-TB in Lesotho. Although the XDR-TB in neighborhing KwaZulu-Natal raises concerns about second-line drug resistance in Lesotho, we did not find much (Table 2) [9, 10].

The drug resistance survey conducted in Lesotho had multiple limitations that probably detracted from its reliability. The next drug resistance survey in Lesotho should be undertaken based on the lessons learned from this survey’s implementation, including (a) collecting clinical data on a single form during the first patients visit, (b) transportation of this form to the laboratory along with available sputum samples, (c) consistency in assigning a unique patient DRS identifier, (d) including HIV testing as per national guidelines, (e) planning for hazardous weather conditions, (f) repeat trainings throughout the survey operations with regular supervision and monitoring of DRS processes, and (g) real time electronic data entry to facilitate ongoing enrolment at diagnostic facilities.
